# Reduction of social inequalities in life expectancy in a city of Southeastern Brazil

**DOI:** 10.1186/1475-9276-10-36

**Published:** 2011-08-26

**Authors:** Ana Paula Belon, Marilisa BA Barros

**Affiliations:** 1 Department of Preventive and Social Medicine, School of Medical Sciences, State University of Campinas, São Paulo, Brazil

## Abstract

**Background:**

Around the world the life expectancy at birth has risen steadily over time. However, this increase in life years is not equally distributed among different social segments of the population. Studies have demonstrated that social groups living in deprived areas have a shorter life expectancy at birth in comparison to affluent ones. The aim of this study was to evaluate inequalities in life expectancy by socioeconomic strata in a city with one million inhabitants in Southeastern Brazil, in 2000 and 2005.

**Methods:**

Through an ecological approach, the 49 areas of health care units of the city were classified into three socioeconomic strata, defined according to variables of income and educational level of the heads of household obtained from the 2000 Census. Life tables were constructed by sex for each of the three socioeconomic strata in 2000 and 2005.

**Results:**

The life expectancy at birth for men and women living in poor areas was 6.9 and 5.5 years lower in comparison to the affluent ones in 2000. Between 2000 and 2005, these social inequalities in life expectancy at birth reduced, since the groups with lower socioeconomic level had gained more life years. The increase in life expectancy at birth experienced by areas with worse living conditions was 3 times higher than the increment estimated for prosperous areas for both sexes. Males had the greatest gain in life years, leading to a narrowing of gender differentials in life expectancy between 2000 and 2005.

**Conclusions:**

The reduction of social inequalities in life expectancy suggests that living and health conditions have improved over time, due to social and health policies. The expansion of both health care coverage and cash transfer policies could have had positive effects on mortality reduction and on the consequent increase in the life expectancy, especially for the poor population.

## Introduction

Around the world the trend in life expectancy at birth has been characterized by a steady increase [1,2]. However, the gain in life years is not equally distributed among the different social segments of the population [3-5]. Studies have found wide social inequalities in life expectancy at birth and in the gain in life years, according to the position or type of occupation, educational level, and income of population subgroups [6,7].

Although there is no consensus on the existence of association between social inequalities and mortality [8-14] and on the intensity of its effects in countries with larger or smaller social inequality levels, as well as on whether these inequalities would be mortality predictors [14-16], several studies have demonstrated that social groups in worse living conditions present a lower life expectancy at birth [3,5-7,17-19].

Concerning the investigations about the trend of social inequalities in life expectancy, several studies have indicated increasing disparities [3,6,13], while others have pointed to reduction [20]. Compared to the international scientific literature, not many studies in Brazil have approached the relation between socioeconomic inequality and mortality [18,21,22]. And just a few of them investigated the impact of this social inequality on life expectancy [23,24], despite the capacity of this indicator to summarize the mortality level and express living and health conditions of the population.

The use of indicators for monitoring social inequalities in health is still highly relevant, since it subsidizes the assessment of public policies and the definition of priority programs [21], so as to promote equity in health.

Therefore, the aim of this study was to evaluate inequalities in life expectancy by socioeconomic strata in the city of Campinas, Brazil, in the years 2000 and 2005.

## Methods

This study was carried out in Campinas, a major economic pole in the state of Sao Paulo and the fifth city in terms of the contribution to the state's Gross National Product. Located 100 Km from the state capital, Sao Paulo city, Campinas has an expressive industrial park and it is the home to major Brazilian research centers, including the second most important Brazilian university, State University of Campinas (UNICAMP), and one of the main public hospitals in the country, the State University of Campinas Hospital. With a population of 968,157 inhabitants in 2000 and 1,080,113 in 2010, Campinas is the 14^th ^most populous city in Brazil.

Despite the poor quality of mortality data in some Brazilian states, the vital statistics data are reliable in the State of Sao Paulo. According to the Inter-Agency Health Information Network^1^, an organization created by Brazilian Ministry of Health and Pan American Health Organization, the coverage of death registration was estimated at 97.7% and the proportion of deaths assigned to ill-defined causes was only 6.4% in 2007. Campinas also has high quality vital registration systems. From the total of causes of death recorded in this city, less than 3% are assigned to the ill-defined codes. In addition, in Campinas there is a project that monitors the mortality in the city since 1989. Through of a partnership between the Municipal Health Department of Campinas and the State University of Campinas, this project guarantees improvement in the data quality, eliminating problems related to the completeness and accuracy in the vital statistics sources of this city.

This descriptive study used secondary data for the construction of abridged life tables by sex for each of the three socioeconomic strata of the population resident in Campinas in 2000 and 2005. Through an ecological approach, the socioeconomic strata were defined according to variables of income and educational level of the head of household obtained from the 2000 Census of the Brazilian Institute of Geography and Statistics (IBGE). Census data are available for the 49 areas of health care units at the Municipal Health Department in Campinas. Through the geocoding techniques, the Municipal Health Department of Campinas performed the identification of census tracts in each of these 49 areas. The socioeconomic stratification of the health care unit areas was based on the following variables: the percentage of heads of household that earned equal to or more than 10 minimum wages per month, the percentage that earned less than 2 minimum wages per month, the percentage of heads of household with 10 or more years of schooling and the percentage with less than 1 year of schooling. A global score was set for each area of health care unit, considering the average of its position in each of the four indicators. Using this global score, these areas were sorted and grouped into three socioeconomic strata: High, Medium and Low. With a population of around 33.3% of the city total, each stratum was defined as a homogeneous socioeconomic area. The stratification outlined with this technique presented similar results as the ones obtained with Cluster analysis, which used the hierarchical method of Ward/SAS 2002 with agglomerative function [25].

The information about the population residing in the city in 2000 was provided by the Municipal Health Department (MHD). With data from the 2000 Census, MHD redistributed the population size according to the health care unit areas, using the data from the corresponding census tracts. Based on the 1991 and 2000 Census data, population estimates for Campinas and the areas served by the health care units for the year 2005 were calculated. The MHD provided these estimates using the AiBi method. This method allows the calculation of projections for small areas, adopting the population growth of the larger area as a parameter [26].

The vital statistics were collected from MHD, which codifies these events according to the areas encompassed by the health care units, using the home address of the individual. Data on infants born-alive by sex and residence areas defined by the health care units for the years 1999-2001 and 2004-2006 were gathered from the Live Births Information System (SINASC). Deaths by sex, age group, and residence area were obtained from the Mortality Information System (SIM). Due to under-enumeration in mortality data from the MHD in the 1990s and at the beginning of 2000s, the total number of deaths recorded in the Information Technology Department of the Unified National Health System (DATASUS) from the Brazilian Ministry of Health was used for the years 1999 to 2001. This total was redistributed according to the mortality age pattern in each socioeconomic stratum, which was calculated using the MHD data for the years 1999 to 2001. Mortality data from MHD were used for 2004-2006.

To calculate age-specific mortality rates for the population above 1 year of age, the triennium average of deaths was used, considering 2000 and 2005 as the central years. The age groups used were 1-4, 5-9, 10-14, 15-19, 20-30... 70-80 and 80 years or more. The infant mortality rates of the two periods analyzed were estimated through the quotient between the number of infant deaths and the number of live births between the years 1999 and 2001 and between 2004 and 2006. Applying Arriaga's method [27], separation factors by sex were calculated for infant deaths in the years 2000 and 2005.

The other life table functions, such as life expectancy at birth (e_0_) and life expectancy at the exact age (e_x_), were obtained based on the conversion of the mortality specific rates by age groups to death probabilities at exact ages (_n_q_x_). The inequalities in life expectancy at birth and at exact ages were measured based on absolute (in years) and relative differences between Low and High socioeconomic strata.

Since this manuscript only used secondary data, ethical clearance was not necessary. Vital statistics and population data provided by Brazilian agencies are freely and universally accessible online.

## Results

Deep social inequalities were observed in the population of Campinas, as shown by the selected demographic and socioeconomic indicators (Table 1). In the demographic composition, we can observe that areas with the lowest socioeconomic status presented a higher percentage of people under 15 years of age (31.1% vs 17.9%) and a lower proportion of elderly (4.9% vs 14.0%) than the most affluent areas. Among the heads of household living in the areas corresponding to the lowest stratum, only 4.4% had income equal to or higher than 10 minimum wages and 13.7% had 10 years of schooling or more. For the higher stratum, these values were 44.5% and 60.4%, respectively.

**Table 1:** Demographic and socioeconomic indicators according to socioeconomic strata and to the population of Campinas in 2000

Indicators	Low	Medium	High	Campinas
Population aged 0-14 years (%)	31.1	22.6	17.9	24.0
Population aged 60 or older (%)	4.9	9.9	14.0	9.5
Households in slum areas (%)	24.9	9.1	1.3	11.1
Heads of household with an average income of less than 2 minimum wages per month (%)	28.6	18.4	9.9	18.1
Heads of household with an average income equal to or more than 10 minimum wages per month (%)	4.4	21.0	44.5	25.2
Heads of household with less than 1 year of schooling (%)	9.8	5.9	2.5	5.9
Heads of household with 10 or more years of schooling (%)	13.7	33.8	60.4	37.3

Total population in 2000*	329567	324797	313793	968157

Total population in 2005*	386114	332666	309819	1028599

Source: 2000 Demographic Census/Brazilian Institute of Geography and Statistics (IBGE).

* Population data by Municipal Health Department (MHD).

In the population of the High socioeconomic stratum, the life expectancy at birth was the highest in the two years studied: 75.2 years in 2000 and 76.5 years in 2005 (Table 2). The shorter life expectancy at birth occurred in the population of the Low socioeconomic stratum (68.7 years in 2000 and 72.3 years in 2005). Life expectancy was higher for women than for men in the three socioeconomic strata and in both years studied. However, the greatest gains in life years during this period occurred among men in the three socioeconomic strata.

**Table 2:** Life expectancy at birth by sex and socioeconomic strata. Campinas, 2000-2005.

Socioeconomic strata	Life expectancy at birth		Variation between 2000 and 2005	Differences in life expectancy (years) among strata (reference category: High)	
	2000	2005		2000	2005
**Males**					
**High**	71.1	73.2	2.1	-	-
**Medium**	68.1	70.4	2.3	-3.0	-2.8
**Low**	64.2	68.9	4.7	-6.9	-4.3
**Females**					
**High**	79.3	79.6	0.3	-	-
**Medium**	77.7	77.6	0.0	-1.6	-2.0
**Low**	73.8	76.0	2.2	-5.5	-3.6
**Both sexes**					
**High**	75.2	76.5	1.3	-	-
**Medium**	72.7	73.9	1.2	-2.5	-2.6
**Low**	68.7	72.3	3.6	-6.5	-4.2

Comparing the variation in life expectancy at birth between 2000 and 2005 according to the socioeconomic strata, it was possible to observe that the areas with worse living conditions experienced the largest increase in life expectancy. In these areas, the life expectancy at birth increased 4.7 years for men and 2.2 years for women during the study period. Among women, only the Medium stratum had no record of increase in the life expectancy at birth, and in the High stratum the increase was very small (Table 2).

The greatest inequalities in life expectancy at birth among socioeconomic strata were recorded among men in both years analyzed. In 2000 the gap between High and Low socioeconomic strata reached a value of approximately 7 years. Among females, this difference was 5.5 years in 2000. From 2000 to 2005 inequalities between these two strata were reduced to 4.3 years for males and to 3.6 years for females (Table 2).

**Figure 1:**
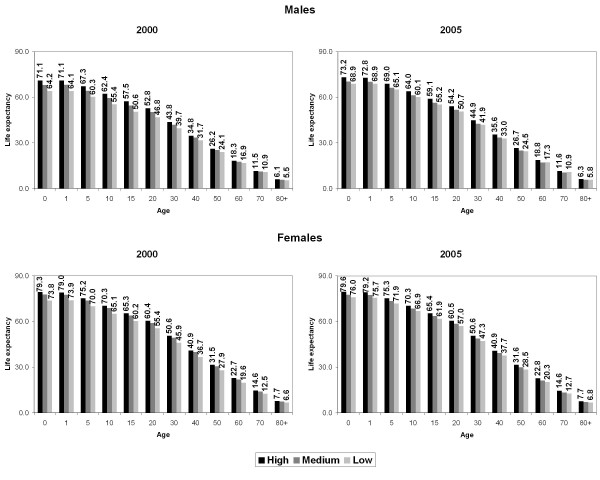
**Life expectancy at exact age by sex and socioeconomic strata**. **Campinas, 2000-2005.**

**Figure 2:**
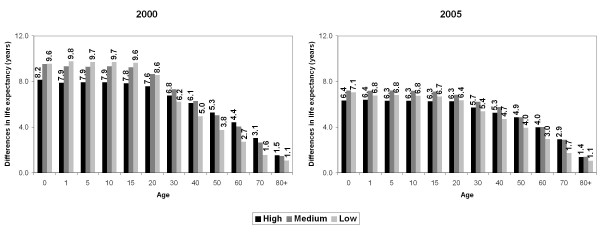
**Gender differences in life expectancy at exact age, by socioeconomic strata**. **Campinas, 2000-2005.**

The social pattern of life expectancy at exact age did not differ from those verified in the previous table: at each age the values decreased from High to Low socioeconomic strata and the women experienced the greatest life expectancy in all ages (Figure 1). In the analysis of gender differences in the life expectancy, different patterns were observed according to each age (Figure 2). The analysis of the extreme strata (High and Low) shows that, in 2000, the Low stratum presented the greatest gender inequalities in life expectancy at birth until the age of 20. The gap between men and women was around 10 years. After age 30, the widest gender differences were recorded in the highest socioeconomic stratum. This age pattern of gender inequalities remained in 2005. However, the magnitude of these gender differences in each socioeconomic stratum decreased between 2000 and 2005. For instance, the life expectancy at birth declined from 8.2 years to 6.4 years in the High stratum and from 9.6 years to 7.1 years in the Low stratum.

**Figure 3:**
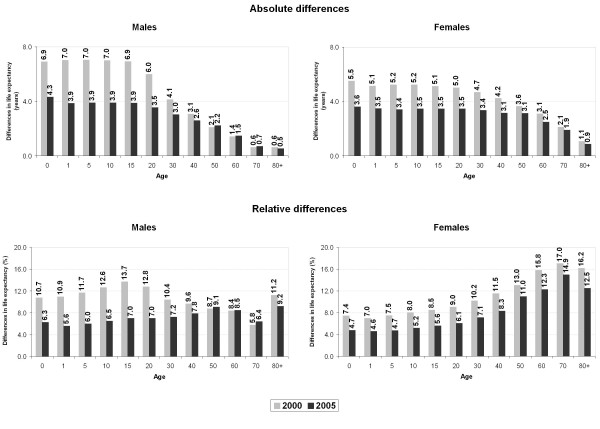
**Differences in life expectancy between High and Low socioeconomic strata, by sex**. **Campinas, 2000-2005.**

Absolute and relative differences in life expectancy between Low and High strata for each sex in 2000 and 2005 are depicted in Figure 3. In the two years studied and for both sexes the largest absolute differences were concentrated in the younger age groups, with the gap decreasing consistently with age. Moreover, the absolute differences in the life expectancy were greater among men until age 20, while the social inequalities in this indicator were larger among females from the age of 30. From 2000 to 2005 the inequalities in life expectancy between High and Low socioeconomic strata declined in both sexes and in almost every age studied. But this reduction was more pronounced among young men: in 2000, while the male life expectancy at age 15 in the Low stratum was 6.9 years shorter than that in the most affluent areas, in 2005 this difference was only 3.9.

Analyzing relative differences in male life expectancy between the two extreme socioeconomic strata, we observe that the greatest inequalities were concentrated among young people (Figure 3). In 2000, the relative differences in the younger age groups were around 12%. Among females, in contrast to the gradient observed in absolute indices, the inequalities were more expressive, in percentage terms, in the older ages. In other words, although the absolute differences in female life expectancy between socioeconomic strata were smaller in older ages, in relative terms the inequalities were larger in these age groups when compared to younger ages. In 2000, for example, the absolute difference in the female life expectancy at age 70 between the extreme socioeconomic strata was only 2.1 years. However, in percentage terms, this difference represented a distance of 17.0% between the strata-the largest index recorded for the female population. In 2005, an increasing tendency in these differences was observed with aging, which was more evident among females. Between 2000 and 2005 there was a reduction in the relative differences in almost every age for both sexes.

## Discussion

The findings of this study reveal a gradient in the life expectancy according to living conditions of the population by area of residence, expressing the impact of social factors on health. Although the life expectancy at birth in Campinas, estimated at 74.7 years for 2005 [28], is higher than the average for Brazil and for the state of Sao Paulo [29], there are important differences in the average life span in the city, due to unequal living conditions.

Our results were produced from a regional mortality analysis using aggregate data since the national vital statistics system in the country presents deficient socioeconomic information, such as education, occupation, and race. The incompleteness of these variables in the mortality database [30] prevents their application for the establishment of socioeconomic strata and consequent exploration of this relationship in an individual level. Therefore, we used the Census data of the year 2000. Since our unit of analysis is the residence area, a limitation of this study is that our results do not necessarily reflect the situation of individuals that belong to each socioeconomic stratum. Furthermore, it is known that the use of aggregate level information has limitations because it presents the average of indicator [11,31]. In Campinas, several health care units serve a very large geographic area or have to cope with significant social inequalities in the same area. However, the adoption of health care unit areas as spatial units of social stratification provides important advantages in terms of public health policy actions. The construction of indicators for these political-administrative units allows for the assessment of their health actions and interventions.

With the socioeconomic stratification of health care unit areas, sharp inequalities in life expectancy at birth and at exact ages were identified. The areas corresponding to the stratum of low socioeconomic level, as expected, presented the shortest life expectancies in 2000 and 2005. Similar results were verified by Szwarcwald et al. [18] in an ecological study in Rio de Janeiro that pointed out, in 1991, that areas with more poverty concentration had the shortest life expectancy at birth. In the most deprived areas of Rio de Janeiro, life expectancy was 9 years lower than the municipal average.

Nevertheless, in Campinas, an important finding was that social inequalities in life expectancy at birth reduced between 2000 and 2005. In 2000, a newborn from areas with poorer living conditions could expect to live on average 6.5 years less than a child from wealthier areas. In 2005, this difference decreased to 4.2 years.

In some countries, studies have found a rise in inequalities in the life expectancy among different socioeconomic groups [3,6,7,17,19,32]. A study in New Zealand [7] pointed out an increase in income inequality as the main explanation for the rising disparities in life expectancy among the Health Districts Boards in that country between 1980 and 2001, which among males increased from 2.3 years to 3.8 years. In a study about life expectancy disparities among counties in the United States, which were categorized by a factor-based deprivation index, Singh and Siahpush [6] found that the socioeconomic indicators have generally improved during the study period. Nevertheless, the social gradient among these groups remained stable and the gap in life expectancy at birth increased from 2.8 years to 4.5 years between 1980 and 2000, since the groups with higher socioeconomic level gained more years of life.

In Campinas the reduction of social inequalities in life expectancy is due to a significant increase in life years in areas corresponding to lower socioeconomic stratum, which was 3 times higher than that estimated for prosperous areas for both sexes. In male and female populations, the highest gains in these poorer areas represented an increment of 4.7 and 2.2 years, respectively. Duarte et al. [23] also observed that Brazilian states with the lowest life expectancy at birth were precisely those that presented the highest gain in life years between 1991 and 1999. This relation was statistically significant in the case of the male population. However, international studies that verified an increase in this difference in life expectancy pointed out that the greatest gains in life years occurred in the richest areas or among people with higher living conditions [3,6,7,32]. Brønnum-Hansen and Baadsgaard [3], analyzing the increment of life years in males aged 30 and older during the period 1996 to 2005 in Denmark, estimated that gains in the life expectancy were only 0.73 for men with a low educational level, while there was an increase of 1.06 years for people with higher levels of education. Similarly, in the United States, between 1990 and 2000, groups with a high educational level experienced larger gains in life expectancy at age 25, while, in the other group, the index remained unchanged [13]. In Lithuania, Kalėdiėnė, Starkuvienė and Petrauskienė [17] verified that people with lower educational levels had a loss of life years, mainly due to external causes. This situation led to a decrease in life expectancy between 1989 and 2001.

This study still reveals the magnitude of social inequalities regarding life expectancy for each sex. In Campinas, as in some countries [7,13,32], men experienced the largest gains in life expectancy at birth during the period analyzed. The main reason for this increase was a significant decline in the homicide rates in Campinas, which was also registered in other cities in the state of Sao Paulo at the beginning of 2000s. According to Belon and Barros [28], reductions in mortality rates from external causes have greatly contributed to the increase in male life expectancy at birth, representing 69.7% of the total gain of life years in Campinas between 2000 and 2005. Associated to this increase in male life expectancy, the slower growth or even the stagnation of the female average life span allowed the narrowing of gender disparities in life expectancy in the three socioeconomic groups between 2000 and 2005.

Concerning the gender differences in life expectancy, it must be emphasized that the reduction of these inequalities was more accentuated in the lowest socioeconomic stratum over the period studied. Nevertheless, in 2005, this group still had the widest gender inequalities, compared to wealthier areas, with 7.1 years and 6.4 years respectively. Singh and Siapush [6] and Raleigh and Kiri [32] also found that the largest gender difference occurred in regions with the worst deprivation index. In the case of England and Wales [32], gender differences in the most- and least-deprived Health Districts varied from 6.6 to 5.4 years.

The findings also reveal another dimension of social inequalities in relation to life expectancy between males and females. Between the extreme socioeconomic strata, the gaps in life expectancy at birth until life expectancy at age 20 were the largest among males. For example, in 2000, a male newborn from the areas corresponding to the worst socioeconomic stratum could live on average 7 years less than a child from areas of the High stratum, while this difference among females was 5.5 years.

In most countries, it is possible to observe that female life expectancy is higher than the male one. However, it is interesting to observe that, as registered in England [32] and also in Campinas in 2000 and 2005, male life expectancy in the affluent areas is lower than the female one in the areas with the worst living conditions.

In other investigations that have also verified these gender differences in life expectancy and in other mortality indicators, some hypotheses were elaborated to explain why the impact of socioeconomic inequalities is higher for males than for females [6,10,22,32,33]. A first explanation would be that disadvantaged living conditions would be associated with the adoption of unhealthy behaviors (smoking and excessive alcohol consumption, for example), which are more prevalent among males than among females. These harmful behaviors would be risk factors for diseases and injuries (such as external causes, respiratory and hepatic diseases), which, in turn, have a greater impact on male mortality, particularly on premature mortality indicators [10,32]. In other words, gender differences in health-related behavior patterns could partially explain gender differences in the mortality pattern. And although an approximation is occurring between the sexes concerning the unhealthy behaviors, women would be prone to adopt them with more moderation and would seek health care services more regularly [34]. Another explanation, complementary to the previous one, would be that greater incidence of accidental and violent deaths occurs in areas of lower socioeconomic levels [22,32]. This could explain why men with lower socioeconomic levels have shorter life expectancy than men residing in more affluent areas. In addition, there is a psychosocial interpretation in the literature which states that females would be less vulnerable to adverse social conditions and that the male mortality rates would be more sensitive to the socioeconomic context [33].

In Campinas, previous studies have demonstrated that social inequalities manifest themselves in natural, as well as in accidental and violent causes of death [28]. These studies also indicate that mortality among males is higher than among females [35]. Based on these data, it becomes evident that the male population resident in the areas with worse living conditions is the group most exposed to the external causes, either compared to men from the highest socioeconomic stratum or to women who live in the most disadvantaged areas.

It is interesting to examine that from the age of 30, the gap in the life expectancy among strata became larger among females. One reason would be that external causes, which are the main determinant of premature mortality among males, would become less frequent from the age of 30. Besides this, another possible explanation would be that although health indicators for females are better than for males, there is an accumulation of disadvantageous situations along the lifetime among poorer females, which appear in adulthood and old age. These situations are responsible for disparities in the life expectancy between higher and lower socioeconomic level areas.

Despite the narrowing socioeconomic inequalities in life expectancy between 2000 and 2005, the disparities still remain high. These social disparities may be more related to different lifestyles according to the socioeconomic stratum. The groups with the worst living conditions would be more exposed to risk factors for several diseases, such as physical inactivity, excessive alcohol consumption, obesity and inappropriate diet [3,6,17,36,37]. Cockerham [36] adds to this discussion the possession of resources, viewed as an accumulation of means to afford choices and chances in lifestyle, explaining that it is easier for people with better socioeconomic conditions to adopt healthy behaviors. Thus, interventions in the health area aimed at behavioral changes have great possibilities of reducing social inequalities in health, although public policies focused on ensuring equity in living conditions are even more important, considering that social inequalities are the main determinants of disease and premature mortality [5,6].

The reduction of social inequalities in the average life span in Campinas between 2000 and 2005 suggests not only an improvement in mortality rates, but also shows that measures such as the expansion of both health care coverage and cash transfer policies are ensuring better living and health conditions [38-40]. Certainly, these measures have positive effects on mortality reduction and on the increase in the life expectancy at birth, especially for the poor population.

In Brazil, there are many policies that although not directly related to the improvement in health conditions, may have contributed to the decline in mortality rates with consequent increase in life years. These include public policies of cash transfer, programs focused on providing more access to the education system (from primary school to university) such as adult alphabetization, and programs creating incentives for the permanence in school, in addition to food security programs (such as anti-hunger programs) and social protection policies.

For the period analyzed here some investigations about cash transfer programs reported that they exerted a great impact on reducing poverty and social inequalities [38]. These programs promoted the decline in income inequality and an increase in the average income of the poorer population between 2000 and 2005 [39]. Recent diagnostics have indicated the expansion of cash transfer program coverage with expressive positive impacts on the reduction of income inequalities and on the access and permanence in primary school [40]. This may contribute even more to the narrowing of gap in life expectancy among different social groups. Other aspects to be considered about the cash transfer programs and their role in reducing inequalities in health are the obligations imposed, which are not restricted to the education sector, but are extended to health and food security, including the vaccination scheme, pre-natal care, and monitoring child development with food and nutrition surveillance. Although there are not many studies that assess the impact of cash transfer programs on health [41], the delivery of monetary resources and the accomplishment of counterparts stipulated by the programs have promoted greater access to health services and nutritional well-being, leading to improvements in general health conditions.

Another important factor that may explain the decline of social inequalities in mortality are health policies and programs. Shi et al. [42] state that, although less investigated by several health determinant models, the health care system would interact with contextual variables of income concentration, leading to a reduction of adverse effects on health produced by social inequalities. In Brazil, the expansion of the Family Health Program represents an important step towards the decline of social inequalities in primary health care access [43]. The availability of access to public health services of the Brazilian National Health System (SUS) in Campinas may have partially compensated for the adverse effects of social inequalities, reducing mortality levels of population with lower socioeconomic status, which is the group most dependent on the public health system. The expansion of availability of specific programs aimed at promoting health and preventing diseases and risk factors, associated to greater health assistance with the expansion of the Family Health Program coverage, can have favored the decline in mortality levels. This can be seen mainly in relation to the social groups with worse living conditions, enabling a smaller difference in the average life span among different socioeconomic groups.

## Conclusions

These findings clearly demonstrate the impact of socioeconomic inequalities on the mortality, indicating that population living in poor areas present a shorter life expectancy in comparison to the affluent ones.

The results of this study also pointed out that the population resident in the most disadvantaged areas experienced larger gains in life years during the study period, leading to a decline in social inequalities in life expectancy. Possibly, the reduction of socioeconomic inequalities in Brazil due to the expansion of public policies coverage, such as cash transfer programs, associated to the increase in health care coverage, may have contributed to this narrowing of social disparities in the average life span. Another important factor was the significant decline in homicide rates in Campinas at the beginning of 2000s, leading to the narrowing of socioeconomic inequalities in male life expectancy.

The reductions of disparities in life expectancy among socioeconomic strata indicate possibilities of ensuring the population more equity in health conditions. It is worth highlighting that these results differed from those observed in some developed countries. Several studies found widening social inequalities in life expectancy in countries with a more egalitarian income distribution.

Therefore, this study can contribute to the development of health actions and policies elaborated according to the reality of each socioeconomic group, aiming to further reduce social inequalities in mortality.

## Competing interests

The authors declare that they have no competing interests.

## Authors' contributions

APB and MBAB have made substantial contributions to conception of the article and to analysis and interpretation of data. Both authors have read and approved the final manuscript.

## Notes

^1^http://tabnet.datasus.gov.br/cgi/idb2009/matriz.htm
